# Risk of Depression in the Adolescent and Adult Offspring of Mothers With Perinatal Depression: A Systematic Review and Meta-analysis

**DOI:** 10.1001/jamanetworkopen.2020.8783

**Published:** 2020-06-30

**Authors:** Vaishali Tirumalaraju, Robert Suchting, Jonathan Evans, Laura Goetzl, Jerrie Refuerzo, Alexander Neumann, Deepa Anand, Rekha Ravikumar, Charles E. Green, Philip J. Cowen, Sudhakar Selvaraj

**Affiliations:** 1 Faillace Department of Psychiatry and Behavioral Sciences, McGovern Medical School, University of Texas Health Science Center at Houston; 2 Centre for Academic Mental Health, Population Health Sciences, Bristol Medical School, University of Bristol, Bristol, United Kingdom; 3 Department of Obstetrics, Gynecology, and Reproductive Sciences, McGovern School of Medicine, University of Texas Health Science Center at Houston; 4 Division of Maternal-Fetal Medicine, Department of Obstetrics, Gynecology, and Reproductive Sciences, McGovern Medical School, University of Texas Health Science Center at Houston; 5 Department of Child and Adolescent Psychiatry/Psychology, Erasmus University Medical Center, Rotterdam, the Netherlands; 6 Lady Davis Institute for Medical Research, Jewish General Hospital, Montreal, Quebec, Canada; 7 Department of Internal Medicine, Mercy Health St Vincent Medical Center, Toledo, Ohio; 8 Center for Clinical Research and Evidence-Based Medicine, Department of Pediatrics, McGovern Medical School, University of Texas Health Science Center at Houston; 9 Medical Sciences Division, Department of Psychiatry, University of Oxford, Oxford, United Kingdom

## Abstract

**Importance:**

Maternal depression during pregnancy is associated with emotional and behavioral difficulties of offspring during childhood that can increase the risk of depression in adolescence and adulthood.

**Objective:**

To investigate the association between perinatal maternal depression and an increased long-term risk of depression in their adolescent and adult offspring.

**Data Sources:**

A systematic search of the electronic databases of PubMed and PsycINFO was conducted from May 2019 to June 2019.

**Study Selection:**

A total of 6309 articles were identified, of which 88 articles were extracted for full-text review by 2 reviewers. Only articles reporting data from prospective longitudinal studies that assessed maternal depression during antenatal and/or postnatal periods and resulting offspring 12 years or older with measures of established psychometric properties were included. Exclusion criteria consisted of all other study designs, mothers with other medical and psychiatric comorbidities, and offspring younger than 12 years.

**Data Extraction and Synthesis:**

Data were extracted by 2 independent reviewers, and discrepancies were mediated by an expert third reviewer. Meta-analysis was performed using Bayesian statistical inference and reported using Meta-analysis of Observational Studies in Epidemiology (MOOSE) guideline. The association of depression timing with the sex of offspring was explored using metaregression.

**Main Outcomes and Measures:**

Offspring depression was evaluated using standardized depression scales or clinical interviews.

**Results:**

Six studies with a total of 15 584 mother-child dyads were included in the meta-analysis, which found the offspring of mothers who experienced perinatal depression to have increased odds of depression (odds ratio [OR], 1.70; 95% credible interval [CrI], 1.60-2.65; posterior probability [PP] [OR >1], 98.6%). Although metaregression found no evidence for an overall association between perinatal depression timing and offspring depression (antenatal vs postnatal, PP [OR >1] = 53.8%), subgroup analyses showed slightly higher pooled odds for the antenatal studies (OR, 1.78; 95% CrI, 0.93-3.33; PP [OR >1] = 96.2%) than for the postnatal studies (OR, 1.66; 95% CrI, 0.65-3.84; PP [OR >1] = 88.0%). Female adolescent offspring recorded higher rates of depression in metaregression analyses, such that a 1% increase in the percentage of female (relative to male) offspring was associated with a 6% increase in the odds of offspring depression (OR, 1.06; 95% CrI, 0.99-1.14; τ^2^ = 0.31).

**Conclusions and Relevance:**

In this study, maternal perinatal depression, especially antenatal depression, was associated with the risk of depression in adolescence and adulthood. More research into the mechanisms of depression risk transmission and assessments of postinterventional risk reduction could aid in the development of future strategies to tackle depressive disorders in pregnancy.

## Key Points

**Question:**

Is maternal perinatal depression associated with increased risk of offspring depression in adolescence and adulthood?

**Findings:**

In this systematic review and meta-analysis that examined 6 prospective longitudinal studies involving 15 584 mother-child dyads, a 70% increase in the odds of adolescent and adult offspring depression was noted among offspring of mothers who had perinatal depression.

**Meaning:**

In this study, maternal perinatal depression was associated with the risk of depression in adolescence and adulthood among offspring; future studies aimed at exploring the neurobiological mechanism of risk transmission and postinterventional risk reduction could improve the management of depressive disorders.

## Teaser

This systematic review and meta-analysis examines the prevalence of depression in adolescent and adult offspring of mothers who experience antenatal and postnatal depression.

## Introduction

Perinatal depression is a depressive episode that occurs in women during pregnancy (antenatal depression) or within 12 months after pregnancy (postnatal depression). Studies show that approximately 10% to 20% of women experience perinatal depression.^1^ Antenatal depression is estimated to affect 7.4% to 12.8% of women, with women in their second and third trimesters being most susceptible.^2^ The prevalence of postnatal depression is estimated to be 10% to 15% of all pregnancies, and it may be as high as 25% in women with low incomes.^3^ Women with severe maternal depression are at an increased risk of self-harm, suicide, substance use disorder, marital difficulties, and parenting difficulties.^4,5,6^ Perinatal depression also affects children by increasing the risk of mental and physical health problems,^7,8^ thus substantially contributing to family and societal burden. The adverse consequences of perinatal depression on childhood development, including increased behavioral difficulties, impaired cognitive ability, and decreased emotional functioning, have been well established.^9,10,11,12^ Studies have noted that additional consequences in adolescent offspring include anxiety, experiencing bullying, and depression.^13,14,15^ Therefore, perinatal depression is a significant health concern as a potentially treatable cause of impaired functioning in offspring.^16,17^

Depression is a heterogeneous condition with environmental factors interplaying with genetic susceptibility in the onset of illness.^18,19^ A 2015 meta-analysis of all twin studies conducted before 2012 reported a modest heritability of 34.4% (95% CI, 30.7%-37.3%) for depressive episodes.^20^ Perinatal depression in mothers may represent a genetic susceptibility to depression, in addition to having a direct environmental consequences on the offspring. A Children of Twins study (CoTs) analysis that examined the covariation between parental and offspring depression attributed the risk transmission to shared environmental factors rather than shared genes alone.^21^ Thus, a substantial part of the risk of transmission is likely due to both environmental and genetic factors between mother and offspring.^22^ Compromised parenting skills, lower socioeconomic conditions, maternal psychiatric comorbidities (particularly postnatal depression), and childhood maltreatment are some of the significant risk factors for offspring depression.^23,24,25,26^ In the case of antenatal depression, the biological stress response has been strongly implicated as a possible mediator of offspring depression.^27,28^ Some studies have reported significant neurodevelopmental changes in the fetus exposed to antenatal depression, especially in the right amygdala,^29,30^ hippocampus, and other corticolimbic areas.^30^ However, the exact mechanism of this risk transmission is still unclear.

Antenatal depression itself is a risk factor for developing postnatal depression.^31,32^ Recent studies on early stress and brain development have brought more attention to the biological basis and causal linkage of the occurrence of emotional and behavioral problems in the offspring.^33^ Elevated levels of peripheral inflammatory markers such as interleukins and C-reactive protein have been reported in both antenatal and postnatal depression.^34,35^ These peripheral inflammatory biomarkers were also positively correlated with hyperactive cortisol secretion in infants immediately after birth and at age 1 year.^36^ Therefore, it is critical to comprehensively examine the cumulative and differential associations of perinatal depression timing with outcomes for offspring.

The estimated risk of adolescent offspring depression associated with perinatal depression varies across different studies and is associated with its timing. Studies^37,38^ have found that offspring of mothers with antenatal depression were 1.3 to 4.7 times more likely to develop depression. A 4.9- to 7.4-fold increase in the risk of developing depression in adolescence^39,40^ has been reported in the case of maternal postnatal depression. Although some studies^37,38,41^ did not report this association, longer follow-up studies using the same cohort data suggest otherwise. Additionally, female offspring of mothers with antenatal depression and male offspring of mothers with postnatal depression are particularly more susceptible to depression according to Quarini et al.^42^ In the same study, the association by sex was not found in children younger than 12 years, suggesting a likely biopsychological process that took effect only after reaching puberty. While postnatal depression is reported to be associated with depression in offspring of mothers with lower educational attainment,^37^ antenatal depression is reported to be an overall stronger factor associated with adolescent offspring depression.^37,39^ Recurrent depressive episodes and the severity of the symptoms in the mother also seem to be associated with the development and prognosis of offspring depression.^43,44,45^

Most studies reporting on the consequences of maternal perinatal depression focus on childhood behavior and development.^46,47,48^ To our knowledge, this study is the first of its kind to systematically review the literature and conduct a meta-analysis of all prospective longitudinal cohort studies that examine the association of maternal antenatal and postnatal depression with offspring depression in adolescence and adulthood. The initial hypothesis of this study was that perinatal maternal depression would be associated with an increased long-term risk of depression in adolescent and adult offspring. This study also explored the differences in depression outcomes with respect to the sex of the offspring as well as the timing of the perinatal depression (ie, antenatal vs postnatal depression).

## Methods

### Search Strategy

A systematic search of the electronic databases of PubMed and PsycINFO was conducted from May 2019 to June 2019. The search criteria included keyword phrases and different variations for *postnatal depression* OR *antenatal depression* OR *perinatal depression* along with terms for adolescent* and adult offspring* (eAppendix in the Supplement). A validated search filter for prospective longitudinal cohort studies built in collaboration with our liaison librarian was used. We then conducted a secondary search on the reference lists of initially identified studies obtained from the primary search to find additional relevant articles.

The search was limited to studies published in peer-reviewed journals, using human populations. No limitations for language or publication period were set to broaden the scope of the systematic review. Reporting of key characteristics followed the Meta-analysis of Observational Studies in Epidemiology (MOOSE) reporting guideline.^63^

### Study Selection and Data Extraction

**Figure 1.**
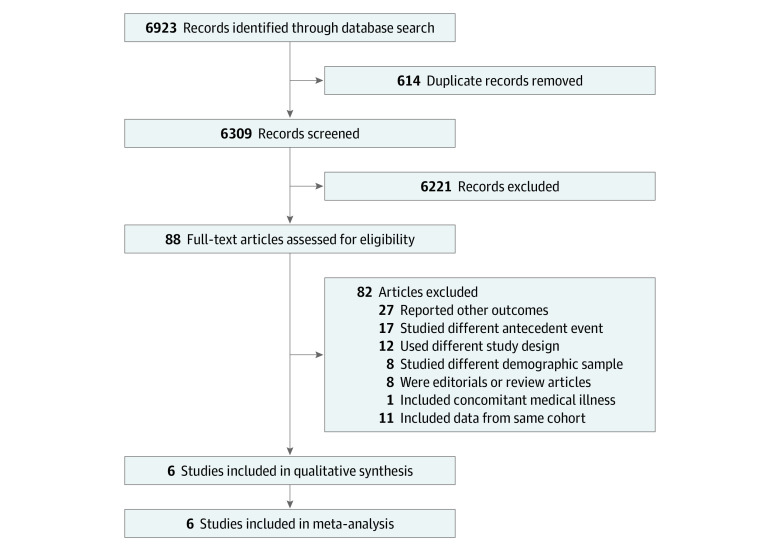
Flowchart of the Study Selection Process

This search strategy returned a total of 6923 studies, with 6309 articles remaining after removing duplicates (Figure 1). Only studies examining associations between maternal depression and adolescent and/or adult depression were included. Titles and abstracts retrieved by the systematic search were independently screened by 2 authors (V.T. and R.R.) using Rayyan QCRI^49^ and filtered according to the inclusion and exclusion criteria. For the purposes of our study, antenatal depression was defined as the presence of depressive symptoms during pregnancy, and postnatal depression was defined as the presence of maternal depressive symptoms within 1 year following delivery.^1^ We contacted authors for additional information if data was unavailable.

We had 4 inclusion criteria when determining eligibility for meta-analysis. First, studies included were prospective longitudinal studies following mothers during their pregnancy and/or during the postnatal period and into the offspring’s adolescence and adulthood. Second, mothers should have been screened for depression during pregnancy and/or at least 1 year after pregnancy. Third, the offspring of mothers not exposed to depression during the antenatal and/or postnatal period were to serve as controls for the study group. Offspring aged 12 years or older were defined as adolescents^50^ and warranted inclusion. Finally, all study outcomes were required to be based on empirical measures with established psychometric properties, such as a standardized depression scale or interview (eg, Edinburgh Postnatal Depression Scale, Standard Psychiatric Interview, Clinical Interview Schedule). Studies that met the following criteria were excluded: cross-sectional studies, retrospective studies, and surveys; mothers with other primary psychiatric or medical comorbidities during pregnancy; and offspring younger than 12 years at the time of assessment for depression. The decision to include or exclude a study was based on a consensus agreement and any discrepancy was mediated by an expert third reviewer (S.S.).

Articles that fit the search criteria underwent full-text review. Upon further filtering out studies using the same cohort information, 6 articles^39,42,43,60,61,62^ were selected for the final review and meta-analysis. The flow of studies through the review selection procedure is shown in Figure 1. The selected studies underwent quality assessment using the Newcastle-Ottawa scale^64^ (eTable in the Supplement).

### Statistical Analysis

Meta-analysis was performed using Bayesian statistical inference in the package brms in the R statistical computing environment version 3.6.2 (R Project for Statistical Computing).^51,52,53,54^ Weakly informative priors (Intercept distributed Normal [0,1]; group-level σ distributed half-Cauchy [0,1]) were chosen for the present analysis to minimize the influence on the meta-analytic estimates per recommendations in the literature.^55,56,57,58^ Assumptions of Bayesian modeling were assessed via posterior predictive checking and convergence diagnostics (eg, effective sample size, chain-mixing convergence).

Effect estimates for the association between maternal and offspring depression were calculated from existing study data using the metafor package in R.^51^ Effect sizes were weighted by their sample variances. In total, 9 effects were calculated from the 6 peer-reviewed articles included in the meta-analysis. Of these, 3 articles provided results for multiple effects via separate results for both antenatal and postnatal maternal depression, 1 article reported data for antenatal depression only, 1 article reported postnatal depression only, and 1 article reported a combined set of values from antenatal and postnatal mothers. Part of the model-fitting process was to determine the extent to which antenatal and postnatal associations could be modeled jointly, and if so, the degree to which depression timing influenced the summary effects. Given that adjusted effects were only provided in 3 of 6 studies and those that did use adjustment included widely different sets of covariates (and thus were not comparable),^59^ the present analysis used unadjusted effects from the raw data in each study.

Pooled log odds and 95% credible intervals (CrIs) were calculated using a multilevel (ie, random-effects) specification, chosen a priori to account for variability across all 9 effects. Log odds were then exponentiated to provide odds ratios (ORs). Given that multiple effects were extracted from 3 of 6 articles, a random intercept for each effect with a hierarchical effect for a paired article identifier was also tested for a superior fit to the data. Goodness-of-fit (leave-one-out cross-validation ) was used to determine the optimal model specification (ie, whether or not the nested intercept improved model fit). A forest plot of Bayesian estimates was generated to visualize the relative contribution of each study to the overall pooled OR as well as the uncertainty within each effect. Metaregression was used to test the potential influence of depression timing and offspring sex (percentage female of offspring sample). Follow-up analyses obtained pooled ORs within each maternal depression timing (antenatal or postnatal). Visual inspection of funnel plot asymmetry and the nonparametric trim-and-fill method were used in the frequentist context (via the metafor package in R) to explore the possibility of publication bias. Individual study influence was investigated via leave-one-out sensitivity analysis, whereby pooled estimates were calculated by omitting each study in turn.

## Results

### Study Characteristics

Our search strategy yielded 6309 studies, out of which 88 articles underwent full-text review, and 11 original articles meeting the inclusion criteria were identified. Upon further filtering out studies using the same cohort information, 6 articles were selected for the final review and meta-analysis. Key characteristics (ie, title, author information, study design, sample size, location, measuring scales, mean ages of offspring at assessment, and number of male and female participants) of the included articles were reported based on the MOOSE guidelines^63^ and are shown in Table 1.^39,42,43,60,61,62^

**Table 1.** Characteristics of Included Studies

Source	Study type	Sample	Sample screening tools	No. of mother-child dyads	Time of maternal perinatal assessments	Maternal measure	Offspring age at analysis, y	No. of offspring		Offspring measure
								Male	Female	
Murray et al,^39^ 2011	Prospective cohort longitudinal study	Cambridge Maternity Hospital, Cambridge, United Kingdom	EPDS, SPI	93	2 and 18 mo postnatally	SPI; Schedule for Affective disorder and Schizophrenia-Lifetime version; SCID	16	45	48	KSADS
Glasheen et al,^60^ 2013	Prospective cohort longitudinal study	Maternal Health Practices and Child Development Project, United States (1982-1985)	CES-D	577	1st trimester, 2nd trimester, during delivery; 8 and 18 mo postnatally	CES-D	16	274	303	DIS-IV
Raposa et al,^61^ 2014	Prospective cohort longitudinal study	Mater Miscericordiae Mother's Hospital-University of Queensland Study of pregnancy, Australia (1981-1984)	DSSI	816	3-4 d and 6 mo postnatally	Average of DSSI Scores at 3-4 d, 6 mo, and 5 y after birth	22-25	413	403	BDI
Plant et al,^43^ 2015	Prospective cohort longitudinal study	South London Child Development Study, United Kingdom (1986)	CIS	103	Antenatally at 20 and 36 wk 3 and 12 mo post birth 4, 11, 16 y post birth	CIS, SADS-L	25	49	54	Structured Clinical Interview for DSM-IV Axis I Disorders, clinical version
Quarini et al,^42^ 2016	Prospective cohort longitudinal study	Avon Longitudinal Study of Parents and Children, United Kingdom (1990-1992)	EPDS	3374	18th and 32nd wk antenatally; 2 and 8 mo postnatally	EPDS; average of 2 antenatal scores and 2 postnatal scores taken as measure for antenatal and postnatal depression respectively	18	1491	1883	CIS-R and ICD-10 criteria for diagnosis
Taka-Eilola et al,^62^ 2019	Prospective cohort longitudinal study	Northern Finland Birth Cohort, Finland (1966)	Nurse interviews enquiring mood	10 521	24th and 28th wk antenatally	Nurse interviews enquiring for mood disturbances	16-43	5395	5126	Diagnoses from the CRHC records validated against DSM-III-R and rereviewed by a professional panel

Abbreviation: BDI, Beck Depression Inventory; CES-D, Center for Epidemiological Studies Depression Scale; CIS, Clinical Interview Schedule; CIS-R, Clinical Interview Schedule-revised; CRHC, Care Register for Healthcare; DIS-IV, Diagnostic Interview Schedule; DSM, *Diagnostic and Statistical Manual of Mental Disorders*; DSSI, Delusions-Symptoms States Inventory; EPDS, Edinburgh Postnatal Depression Scale; ICD, *International Classification of Diseases*; KSADS, Kiddie Schedule for Affective Disorders and Schizophrenia; SADS-L, Schedule for Affective disorders and Schizophrenia; SCID, Structured Clinical Interview for DSM-IV; SPI, Standard Psychiatric Interview.

### Primary Analysis

**Figure 2.**
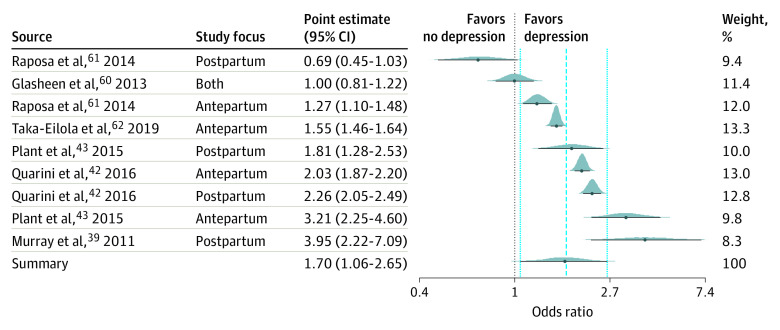
Forest Plot of Summary Odds Ratio Individual curves reflect the posterior distribution for each of the included studies and the summary effect in the meta-analysis. The dotted black line at x = 1 provides the overall cut point for inference. The dashed and dotted lines provide the point estimate and 95% credible intervals for the summary effect.

**Figure 3.**
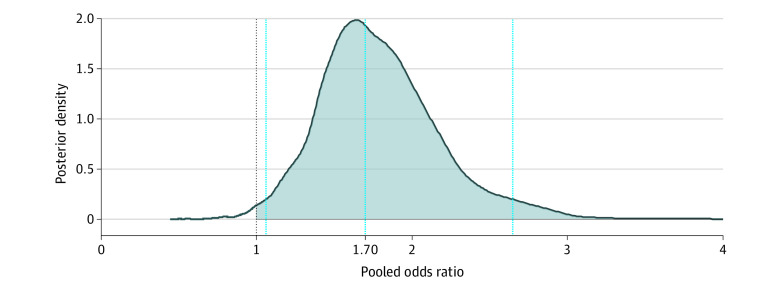
Posterior Distribution for the Summary Odds Ratio The posterior distribution for the summary odds ratio is provided, along with the point estimate (dotted blue line at the median) and 95% credible intervals (dotted blue lines on either side of the median) to describe uncertainty. The dotted black line at x = 1 provides the overall cut point for inference. The shaded portion of the distribution corresponds with the posterior probability that the odds ratio is greater than 1 of 98.6%.

Meta-analysis estimated the pooled OR for the effect of maternal depression on offspring depression including 6 studies (in an overall sample size of 19 535 mother-child dyads). Model specification via leave-one-out cross-validation supported a simple multilevel model above a more complex structure featuring a nested article identifier (ie, only a simple random intercept for each effect was necessary for the present analysis; a nested article identifier for each effect demonstrated worse fit). The best-fitting model yielded a pooled OR of 1.70 (95% CrI, 1.06-2.65; τ^2^ = 0.42), indicating a 70% increase in the odds of offspring depression for mothers who experienced antenatal or postnatal depression (Figure 2). The posterior probability (PP) that the pooled OR was greater than 1 was 98.6%; the posterior distribution for the pooled OR in Figure 3 provides a graphic summary of the association. Bayesian modeling assumptions for the present analysis were satisfied: posterior predictive checking supported the Gaussian outcome distribution for the OR, the scale reduction factor (Rhat) indicated good mixing of MCMC chains, and effective sample sizes were sufficient.

### Metaregression

The association of perinatal depression timing (antenatal vs postnatal) with offspring depression was further explored using metaregression (ie, including a fixed effect of dichotomous timing). This metaregression did not include the study by Glasheen et al^60^ because of a lack of separation between antenatal and postnatal depression. Across the remaining 5 studies (in a sample size of 18 958 mother-child dyads), metaregression did not find strong evidence to support differences between antenatal and postnatal depression, observing only a 53.8% PP that the association of antenatal (compared with postnatal) depression with depression in offspring was greater than 1 (OR, 1.03; 95% CrI, 0.34-3.21; τ^2^ = 0.52).

The percentage of female (relative to male) offspring was also explored using metaregression on all studies. This value was reported in each study and ranged from 40.6% to 55.8% female offspring. Metaregression indicated a 95.0% PP that the effect of percentage female offspring was greater than 1, such that a 1% increase in the percentage of female (relative to male) offspring was related to a 6% increase in the odds of offspring depression (OR, 1.06; 95% CrI, 0.99-1.14; τ^2^ = 0.31). Bayesian modeling assumptions were satisfied for each meta-regression analysis.

### Subgroup Analyses

Follow-up subgroup analyses calculated the pooled OR across studies within each maternal depression period (4 antenatal studies, 14 693 mother-child dyads; 4 postnatal studies, 4265 mother-child dyads). A pooled OR of 1.78 (95% CrI, 0.93-3.33; τ^2^ = 0.31; PP [OR >1] = 96.2%) indicated a 78% increase in the odds of offspring depression for mothers with antenatal depression. A pooled OR of 1.66 (95% CrI, 0.65-3.84; τ^2^ = 1.00; PP [OR >1] = 88.0%) indicated a 66% increase in the odds of offspring depression for mothers with postnatal depression. Bayesian modeling assumptions for both subgroup analyses were satisfied. These subgroup analyses complemented the metaregression analyses: the subgroup analyses found that associations exist for both antenatal and postnatal depression, while the meta-regression indicates that these 2 associations are not different from one another.

### Publication Bias and Influence Analysis

Visual inspection of funnel plot asymmetry did not indicate substantial evidence of publication bias, and the nonparametric trim-and-fill method found no missing studies (eFigure in the Supplement). However, formal testing (ie, Egger test) was not feasible given the small number of measured effect sizes. Omitting individual studies in turn from the meta-analysis (ie, the leave-one-out method) did not change inferences regarding the pooled OR. Influence analyses found a minimum 97.0% PP that the pooled OR was greater than 1 across models, with estimates for the pooled OR ranging from 1.55 to 1.90. This robustness is of particular importance in the present study given the highly discrepant sample sizes across studies; that is, in a multilevel model, the larger sample size studies could have overwhelmed the findings from the smaller studies, but they did not. A summary of the influence analysis is provided in Table 2 (pooled OR, 1.70; 95% CrI, 1.06-2.65).

**Table 2.** Influence Analysis Results

Source	Study focus	OR (95% CrI)	Posterior probability OR >1, %^a^
Murray et al,^39^ 2011	Postnatal	1.55 (0.97-2.27)	97.1
Glasheen et al,^60^ 2013	Both	1.81 (1.12-2.88)	98.8
Raposa et al,^61^ 2014	Antenatal	1.74 (1.01-2.91)	97.8
Raposa et al,^61^ 2014	Postnatal	1.89 (1.26-2.88)	99.6
Plant et al,^43^ 2015	Antenatal	1.55 (0.98-2.41)	97.0
Plant et al,^43^ 2015	Postnatal	1.71 (1.00-2.74)	97.6
Quarini et al,^42^ 2016	Antenatal	1.65 (0.97-2.81)	97.0
Quarini et al,^42^ 2016	Postnatal	1.63 (1.00-2.69)	97.6
Taka-Eilola et al,^62^ 2019	Antenatal	1.72 (0.98-2.89)	97.2
Pooled OR across all	NA	1.70 (1.06-2.65)	98.6

Abbreviation: CrI, credibile interval; NA, not applicable; OR, odds ratio.

^a^Posterior probability OR is greater than 1 when each study was left out of the meta-analysis.

## Discussion

The results of the primary analysis found a 70% increased odds of depression in the offspring of mothers who experienced maternal depression during pregnancy. It supports the initial hypothesis that a maternal history of antenatal or postnatal depression is associated with an increased risk of offspring depression in adolescents and adults. Although metaregression analyses suggested that depression timing was not associated with model outcomes, subgroup analyses within each time (antenatal and postnatal) found a slightly higher pooled OR for the antenatal studies (78% increase in the odds of depression) than the postnatal studies (66% increase).

A recent review^80^ reported that while 50% of women with antenatal depression and 30% of women with postnatal depression are diagnosed in the clinics, only 6% to 8% of them receive adequate treatment. Maternal depression during and after pregnancy has been associated with reduced growth rates, malnutrition, an increased risk of asthma and atopic diseases, childhood obesity, infections, and maltreatment in infancy and childhood.^65^ In addition to the poor physical health outcome itself being an independent risk factor, persistent depression in mothers increases the risk of early-onset depression in the offspring as a result of poor engagement and impaired ability to meet the child’s needs.^66^ One of our included studies (Plant et al^43^) reported on a direct trajectory between perinatal depression and childhood maltreatment as a mediating link for depression in young adults. While most teenagers recover from the first episode of depression, following recovery they are found to have a progressive or recurrent course into adulthood.^67,68^ Repeated or concurrent exposure to episodes of depression during adolescence significantly affects the chronicity of their illness.^69^ Therefore, targeted early interventions at different phases of development of depression will be required to reduce the cumulative effect of maternal depression, early life stressors, and repeated depressive episodes.

Although the exact mechanism remains unclear, newer early life stress models in animals have shown some promising leads in understanding this risk transmission. Altered DNA methylation in the promoter regions of *NR3C1* (OMIM 138040), *BDNF* (OMIM 113505), and epigenetic genes such as *CRH* (OMIM 122560), *MECP2* (OMIM 300005),* CNR1* (OMIM 114610),* CRHR2* (OMIM 602034), and* DLGAP2* (605438) have been implicated in the impaired stress response seen in adulthood as a result of maternal separation and maltreatment.^70,71,72,73^ Changes in the *ESR1* gene expression, noted as early as in the first week of life because of lower maternal care, may influence multigenerational changes in maternal behavior and sensitivity.^74^ The increased odds reported in this study may partly reflect the transmission of genetic factors. It would be necessary in future studies to disentangle the extent to which the associations reported between perinatal depression and adolescent offspring psychopathology are environmentally as opposed to genetically mediated, given that the methods and potentials for intervention differ highly depending on the mechanisms involved.

Our findings are also concordant with existing evidence of higher rates of depressive disorders in postpubertal adolescent girls. Several studies have attributed the increased risk of depression to negative body image, poor self-esteem, coping skills, or significant life events as well as possibly genetic heritability.^75,76^ A recent twin studies meta-analysis^33^ reported a depressive episode genetic heritability of about 40% in women (vs 34.4% in men). One study^76^ also suggested that the susceptible genes in female adolescents may affect the long-term stability of a depressive disorder diagnosis in adulthood.

Given the heterogeneous nature of depression, we chose to study depressive symptoms in adolescents and young adults owing to the increased stability in the diagnosis and lesser ambiguity in the symptom spectrum.^77^ This study included only prospective longitudinal studies to minimize recall error, avoid selection bias, and provide more accuracy in establishing a temporal sequence. Furthermore, no evidence for publication bias was found in the included studies.

### Limitations

This study does have some limitations. First, the inadvertent losses to follow-up in the included studies, especially the larger cohort studies, may have contributed to the results. However, studies such as Quarini et al^42^ have countered the issue by imputing the missing data for their primary analyses. While 2 of the remaining studies^39,62^ had very high retention rates for their subjects, 3 others^43,60,61^ reported mean retention rates of 82%, 85%, and 83% respectively. Second, a single standard diagnostic criterion for depression or major depressive disorder in both the mothers and offspring was not specified. For example, Taka-Eilola et al^62^ used a single structured question during antenatal visits for its 1966 birth cohort study due to the absence of specific screening scales at the time. Although this could be a potential limitation, it also makes the study more generalizable by being less dependent on a single diagnostic method. Third, while the lower age limit for inclusion was set at 12 years, the follow-up period varied in each of the studies included. This lack of homogeneity in offspring age at the time of follow-up assessments may have a potential confounding effect, in that studies with longer follow-up may report a higher incidence of depression. Fourth, the number of included studies (6) was relatively low and may inherently limit the precision of the obtained estimates. Fifth, the percentage of mothers with both antenatal and postnatal depression was not available for any of the studies. Therefore, the analysis cannot accurately address the possibility of unreported antenatal depression in a postnatal sample and/or unreported postnatal depression in an antenatal sample. Sixth, it should be noted that the effects of maternal depression may not be specific to that disorder. While participants with comorbid psychiatric diagnoses were excluded, subclinical symptoms of other psychiatric domains may be present and depression may also be representative of general susceptibility to psychopathology.^78,79^ Another possible limitation would be that of unpublished data not identified in our search strategy.

## Conclusions

To our knowledge, this meta-analysis provides the most robust empirical evidence that maternal perinatal depression, especially antenatal depression, could play a substantial role in the onset of depression in adolescence and adulthood. Education about the benefits and risks of psychotherapy and medication approaches should be communicated to patients, families, and clinicians. Given the significant public health consequences of perinatal depression, a constant improvement in our screening efforts and management plans and optimal allocation of resources from public health initiatives are of utmost importance. Further explorative studies of the potential neurobiological and genetic pathways will remarkably improve our understanding of the complexities of depressive disorders. Specific studies designed to ascertain the evidence of postinterventional risk reduction in depression will aid in the development of future strategies to tackle this serious public health problem.

## Notes

Supplement. **eAppendix.** Search Strategy **eTable.** Quality Assessment Using the Newcastle-Ottawa Scale **eFigure.** Fill and Trim Plot
