# Supplementary material for: Risk of Depression in the Adolescent and Adult Offspring of Mothers With Perinatal Depression: A Systematic Review and Meta-analysis
Source: JAMA Netw Open. 2020 Jun 30;3(6):e208783. doi: 10.1001/jamanetworkopen.2020.8783 (PMC7327545; doi:10.1001/jamanetworkopen.2020.8783)
Supplement: Supplement. — eAppendix. Search Strategy eTable. Quality Assessment Using the Newcastle-Ottawa Scale eFigure. Fill and Trim Plot [file jamanetwopen-3-e208783-s001.pdf]

Supplementary Online Content

Tirumalaraju V, Suchting R, Evans J, et al. Risk of depression in the adolescent and adult offspring of mothers with perinatal depression: a systematic review and meta-analysis. *JAMA Netw Open*. 3(6):e208783. doi:10.1001/jamanetworkopen.2020.8783.

eAppendix. Search Strategy

eTable. Quality Assessment Using the Newcastle-Ottawa Scale

eFigure. Fill and Trim Plot

This supplementary material has been provided by the authors to give readers additional information about their work.

eAppendix. Search Strategy

Date search run: 17th June 2019

No limits applied to language and dates.

PUBMED search:

((("Mothers"[Mesh] OR "Postnatal Depression"[Text Word] OR "Depression, Postnatal"[Text Word] OR "Post-Partum Depression"[Text Word] OR "Post Partum Depression"[Text Word] OR "Postpartum Depression"[Text Word] OR "Post-Natal Depression"[Text Word] OR "Post Natal Depression"[Text Word] OR "Antenatal Depression"[Text Word] OR "Ante-Partum Depression"[Text Word] OR "Ante Partum Depression"[Text Word] OR "Antepartum Depression"[Text Word] OR Intra-uterine[Text Word] OR Intrauterine[Text Word] OR "Perinatal Depression"[Text Word] OR "Peripartum Depression"[Text Word] OR "Prenatal Depression"[Text Word] OR "Prepartum Depression"[Text Word] OR "Pre-Natal Depression"[Text Word] OR "Pre Natal Depression"[Text Word]) AND ("Adult Sons"[Text Word] OR "Adult Son"[Text Word] OR "Adult Daughters"[Text Word] OR "Adult Daughter"[Text Word] OR "Adult Offspring"[Text Word] OR "Adolescent Son"[Text Word] OR "Adolescent children"[Text Word] OR "Adolescent daughters"[Text Word] OR "Adolescent daughter"[Text Word] OR "Adolescent Offspring"[Text Word] OR Child[Text Word] OR Children[Text Word] OR "Male child"[Text Word] OR "Female child"[Text Word] OR "Child, Male"[Text Word] OR "Adolescents"[Text Word] OR Adolescence[Text Word] OR Teens[Text Word] OR Teen[Text Word] OR Teenagers[Text Word] OR Teenager[Text Word] OR Youth[Text Word] OR Youths[Text Word] OR "Female Adolescent"[Text Word] OR "Female Adolescents"[Text Word] OR "Male Adolescent"[Text Word] OR "Male Adolescents"[Text Word])) AND ("cohort studies"[MeSH Terms:noexp] OR "longitudinal studies"[MeSH Terms:noexp] OR "follow-up studies"[MeSH Terms:noexp] OR "prospective studies"[MeSH Terms:noexp] OR "retrospective studies"[MeSH Terms:noexp] OR cohort[TIAB] OR longitudinal[TIAB] OR prospective[TIAB] OR retrospective[TIAB]))

PsycINFO search:

#1: "Postnatal Depression".mp. or exp Postpartum Depression/

#2: "Ante-natal depression".mp. [mp=title, abstract, heading word, table of contents, key concepts, original title, tests & measures]

#3: "Antenatal depression".mp. [mp=title, abstract, heading word, table of contents, key concepts, original title, tests & measures]

#4: "Antepartum depression".mp. [mp=title, abstract, heading word, table of contents, key concepts, original title, tests & measures]

#5: "Ante-partum depression".mp. [mp=title, abstract, heading word, table of contents, key concepts, original title, tests & measures]

#6: "Post-natal depression".mp. [mp=title, abstract, heading word, table of contents, key concepts, original title, tests & measures]

#7: "Post-partum depression".mp. [mp=title, abstract, heading word, table of contents, key concepts, original title, tests & measures]

#8: "Peripartum depression".mp. [mp=title, abstract, heading word, table of contents, key concepts, original title, tests & measures]

#9: "Peri-partum depression".mp. [mp=title, abstract, heading word, table of contents, key concepts, original title, tests & measures]

© 2020 Tirumalaraju V et al. *JAMA Netw Open.*

#10: "Perinatal depression".mp. [mp=title, abstract, heading word, table of contents, key concepts, original title, tests & measures]

#11: "Peri-natal depression".mp. [mp=title, abstract, heading word, table of contents, key concepts, original title, tests & measures]

#12: "Prepartum depression".mp. [mp=title, abstract, heading word, table of contents, key concepts, original title, tests & measures]

#13: "Pre-partum depression".mp. [mp=title, abstract, heading word, table of contents, key concepts, original title, tests & measures]

#14: "Prenatal depression".mp. [mp=title, abstract, heading word, table of contents, key concepts, original title, tests & measures]

#15: "Pre-natal depression".mp. [mp=title, abstract, heading word, table of contents, key concepts, original title, tests & measures]

#16: "Adult offspring*".mp. [mp=title, abstract, heading word, table of contents, key concepts, original title, tests & measures]

#17: exp Adult Offspring/

#18: "Adult Son*".mp. [mp=title, abstract, heading word, table of contents, key concepts, original title, tests & measures]

#19: "Adult Daughter*".mp. [mp=title, abstract, heading word, table of contents, key concepts, original title, tests & measures]

#20: "Adolescent offspring".mp. [mp=title, abstract, heading word, table of contents, key concepts, original title, tests & measures]

#21: "Adolescent Son*".mp. [mp=title, abstract, heading word, table of contents, key concepts, original title, tests & measures]

#22: "Adolescent Daughter*".mp. [mp=title, abstract, heading word, table of contents, key concepts, original title, tests & measures]

#23: "Male Adolescent*".mp. [mp=title, abstract, heading word, table of contents, key concepts, original title, tests & measures]

#24: "Female Adolescent*".mp. [mp=title, abstract, heading word, table of contents, key concepts, original title, tests & measures]

#25: "Teenager*".mp. [mp=title, abstract, heading word, table of contents, key concepts, original title, tests & measures]

#26: "Teen*".mp. [mp=title, abstract, heading word, table of contents, key concepts, original title, tests & measures]

#27: "Adolescent*".mp. [mp=title, abstract, heading word, table of contents, key concepts, original title, tests & measures]

#28: "Adolescence".mp. [mp=title, abstract, heading word, table of contents, key concepts, original title, tests & measures]

#29: "Youth*".mp. [mp=title, abstract, heading word, table of contents, key concepts, original title, tests & measures]

© 2020 Tirumalaraju V et al. *JAMA Netw Open.*

#30: "Adolescent child*".mp. [mp=title, abstract, heading word, table of contents, key concepts, original title, tests & measures]

#31: ((cohort or longitudinal or prospective or retrospective).ti,ab,id. or longitudinal study.md. or prospective study.md. or retrospective study.md.) not "Literature Review".md.

#32: #1 OR #2 OR #3 OR #4 OR #5 OR #6 OR #7 OR #8 OR #9 OR #10 OR #11 OR #12 OR #13 OR #14 OR #15

#33: #16 OR #17 OR #18 OR #19 OR #20 OR #21 OR #22 OR #23 OR #24 OR #25 OR #26 OR #27 OR #28 OR #29 OR #30

#34: #31 AND #32 AND #33

© 2020 Tirumalaraju V et al. *JAMA Netw Open.*

eTable. Quality Assessment Using the Newcastle-Ottawa Scale.

Study ID	Selection (max 4 stars)	Comparability (max 2 stars)	Outcome (max 3 stars)	Total score
Murray et al (2011)	****	*	***	8
Glasheen et al (2013)	****	*	**	7
Raposa et al (2014)	****	*	**	7
Plant et al (2015)	****	*	**	7
Quarini et al (2016)	****	*	***	8
Taka-Eilola et al (2019)	****	*	***	8

Wells GA, Shea B, O'Connell D et al. The Newcastle-Ottawa Scale (NOS) for assessing the quality if nonrandomized studies in meta-analyses, 2012. Available from: http://wwwohrica/programs/clinical_epidemiology/oxfordasp

Selection:

1) Representativeness of the exposed cohort- a) truly representative of the average adolescent/adult in the community*; b) somewhat representative of the average adolescent/adult in the community*; c) selected group of users; d) no description of the derivation of the cohort

2) Selection of the non-exposed cohort- a) drawn from the same community as the exposed cohort*; b) drawn from a different source; c) no description of the derivation of the non-exposed cohort

3) Ascertainment of exposure a) secure record*; b) structured interview*; c) written self-report; d) no description

4) Demonstration that outcome of interest was not present at start of study a) yes*; b) no

Comparability

1) Comparability of cohorts on the basis of the design or analysis - a) study controls for maternal perinatal depression*; b) study controls for any additional factor*

Outcome

1) Assessment of outcome- a) independent blind assessment *; b) record linkage *; c) self-report ; d) no description

2) Was follow-up long enough for outcomes to occur- a) yes *; b) no

3) Adequacy of follow up of cohorts a) complete follow up - all subjects accounted for *; b) subjects lost to follow up unlikely to introduce bias - small number lost - < 5% *; c) follow up rate < 20% and no description of those lost d) no statement

© 2020 Tirumalaraju V et al. *JAMA Netw Open.*

eFigure. Fill and Trim Plot.

Visual indices of publication bias: funnel plot asymmetry and non-parametric fill-and-trim methods. Plot generated in R package *metafor* via frequentist analogue to the primary Bayesian analysis.

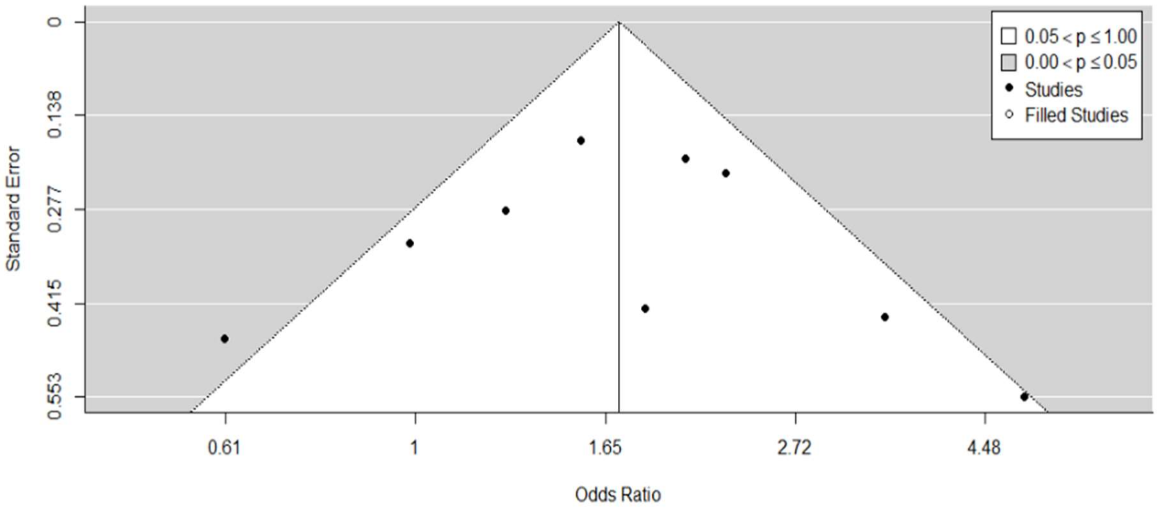

© 2020 Tirumalaraju V et al. *JAMA Netw Open.*
